# Novel *CACNA1C* R511Q mutation, located in domain Ⅰ-Ⅱ linker, causes non-syndromic type-8 long QT syndrome

**DOI:** 10.1371/journal.pone.0271796

**Published:** 2022-07-21

**Authors:** Tadashi Nakajima, Reika Kawabata-Iwakawa, Shuntaro Tamura, Hiroshi Hasegawa, Takashi Kobari, Hideki Itoh, Minoru Horie, Masahiko Nishiyama, Masahiko Kurabayashi, Yoshiaki Kaneko, Hideki Ishii

**Affiliations:** 1 Department of Cardiovascular Medicine, Gunma University Graduate School of Medicine, Maebashi, Gunma, Japan; 2 Division of Integrated Oncology Research, Gunma University Initiative for Advanced Research, Maebashi, Gunma, Japan; 3 Division of Patient Safety, Hiroshima University Hospital, Hiroshima, Hiroshima, Japan; 4 Department of Cardiovascular Medicine, Shiga University of Medical Science, Ohtsu, Shiga, Japan; 5 Gunma University, Maebashi, Gunma, Japan; The Open University, UNITED KINGDOM

## Abstract

**Background:**

Gain-of-function mutations in *CACNA1C* encoding Cav1.2 cause syndromic or non-syndromic type-8 long QT syndrome (LQTS) (sLQT8 or nsLQT8). The cytoplasmic domain (D)Ⅰ-Ⅱ linker in Cav1.2 plays a pivotal role in calcium channel inactivation, and mutations in this site have been associated with sLQT8 (such as Timothy syndrome) but not nsLQT8.

**Objective:**

Since we identified a novel *CACNA1C* mutation, located in the DⅠ-Ⅱ linker, associated with nsLQTS, we sought to reveal its biophysical defects.

**Methods:**

Target panel sequencing was employed in 24 genotype-negative nsLQTS probands (after Sanger sequencing) and three family members. Wild-type (WT) or R511Q Cav1.2 was transiently expressed in tsA201 cells, then whole-cell Ca^2+^ or Ba^2+^ currents (I_Ca_ or I_Ba_) were recorded using whole-cell patch-clamp techniques.

**Results:**

We identified two *CACNA1C* mutations, a previously reported R858H mutation and a novel R511Q mutation located in the DⅠ-Ⅱ linker. Four members of one nsLQTS family harbored the *CACNA1C* R511Q mutation. The current density and steady-state activation were comparable to those of WT-I_Ca_. However, persistent currents in R511Q-I_Ca_ were significantly larger than those of WT-I_Ca_ (WT at +20 mV: 3.3±0.3%, R511Q: 10.8±0.8%, P<0.01). The steady-state inactivation of R511Q-I_Ca_ was weak in comparison to that of WT-I_Ca_ at higher prepulse potentials, resulting in increased window currents in R511Q-I_Ca_. Slow component of inactivation of R511Q-I_Ca_ was significantly delayed compared to that of WT-I_Ca_ (WT-tau at +20 mV: 81.3±3.3 ms, R511Q-tau: 125.1±5.0 ms, P<0.01). Inactivation of R511Q-I_Ba_ was still slower than that of WT-I_Ba_, indicating that voltage-dependent inactivation (VDI) of R511Q-I_Ca_ was predominantly delayed.

**Conclusions:**

Delayed VDI, increased persistent currents, and increased window currents of R511Q-I_Ca_ cause nsLQT8. Our data provide novel insights into the structure-function relationships of Cav1.2 and the pathophysiological roles of the DⅠ-Ⅱ linker in phenotypic manifestations.

## Introduction

Congenital long QT syndrome (LQTS) is an inherited disorder characterized by a prolongation of QT interval and an increased risk of syncope and sudden cardiac death due to polymorphic ventricular tachycardia, torsade de pointes, or ventricular fibrillation [1]. Seventeen genes have been reported to be causal for type-1 to type-17 LQTS (LQT1-17) thus far. However, not all genes appear to be definitely causal for LQTS [2]. Mutations in the first three identified genes—*KCNQ1* for LQT1, *KCNH2* for LQT2 and *SCN5A* for LQT3—account for approximately 90% of genetically affected LQTS patients, while those in other causal genes have rarely been identified [1].

*CACNA1C*, which encodes Cav1.2 composing the pore-forming α-subunit of cardiac L-type voltage-gated calcium channel (I_Ca_), is thought to be definitely causal for LQTS classified as LQT8 [2–4]. Focusing on cardiac disorders, loss-of-function *CACNA1C* mutations have been associated with Brugada syndrome, early repolarization syndrome and short QT syndrome [3, 5, 6]. In contrast, gain-of-function *CACNA1C* mutations have been associated with syndromic LQT8 (sLQT8), namely, presenting with the LQTS phenotype plus other cardiac and/or extra-cardiac phenotypes: Timothy syndrome (TS), an extremely rare disease presenting with QT prolongation along with other cardiac and extra-cardiac phenotypes such as congenital heart defects, autism, developmental abnormalities, neurological dysfunction and syndactyly, atypical TS, and cardiac-only TS (COTS) presenting with QT prolongation along with other cardiac phenotypes such as hypertrophic cardiomyopathy and congenital heart defects but not extra-cardiac phenotypes [7–18]. In addition, gain-of-function *CACNA1C* mutations have also been associated with non-syndromic LQT8 (nsLQT8) presenting with pure LQTS phenotype without other cardiac or extra-cardiac phenotypes [19, 20]. Notably, *CACNA1C* mutations associated with nsLQT8 (pure LQT8 phenotype) have been proven to be more prevalent than previously expected [19, 20]. Although gain-of-function *CACNA1C* mutations can be associated with either sLQT8 (TS, aTS and COTS) or nsLQT8, the precise mechanisms that determine the different phenotypes remain unknown.

We previously analyzed major LQTS-related genes, including *KCNQ1*, *KCNH2*, *SCN5A*, *KCNE1* and *KCNE2*, using Sanger sequencing in patients with nsLQTS in our cohort. However, there remained 24 genotype-negative patients. Therefore, we sought to identify pathogenic variants in these patients using target panel sequencing of 72 genes, including LQTS-related genes [21]. As a result, we identified two *CACNA1C* mutations, including a novel R511Q mutation, but did not detect any other pathogenic variants in LQTS-related genes.

The *CACNA1C* R511Q mutation is located in domain (D)Ⅰ-Ⅱ linker. The segment 6 (S6) in DⅠ (DⅠS6) and the DⅠ-Ⅱ linker play a pivotal role in I_Ca_ inactivation, and *CACNA1C* mutations in the DⅠS6 and DⅠ-Ⅱ linker have been associated with sLQT8 (TS, aTS and COTS) but not nsLQT8 [7, 8, 17, 18, 22, 23]. Therefore, we sought to clarify the biophysical defects of the *CACNA1C* R511Q mutation that causes nsLQT8.

## Materials and methods

### Subjects and genetic analyses

**Fig 1:**
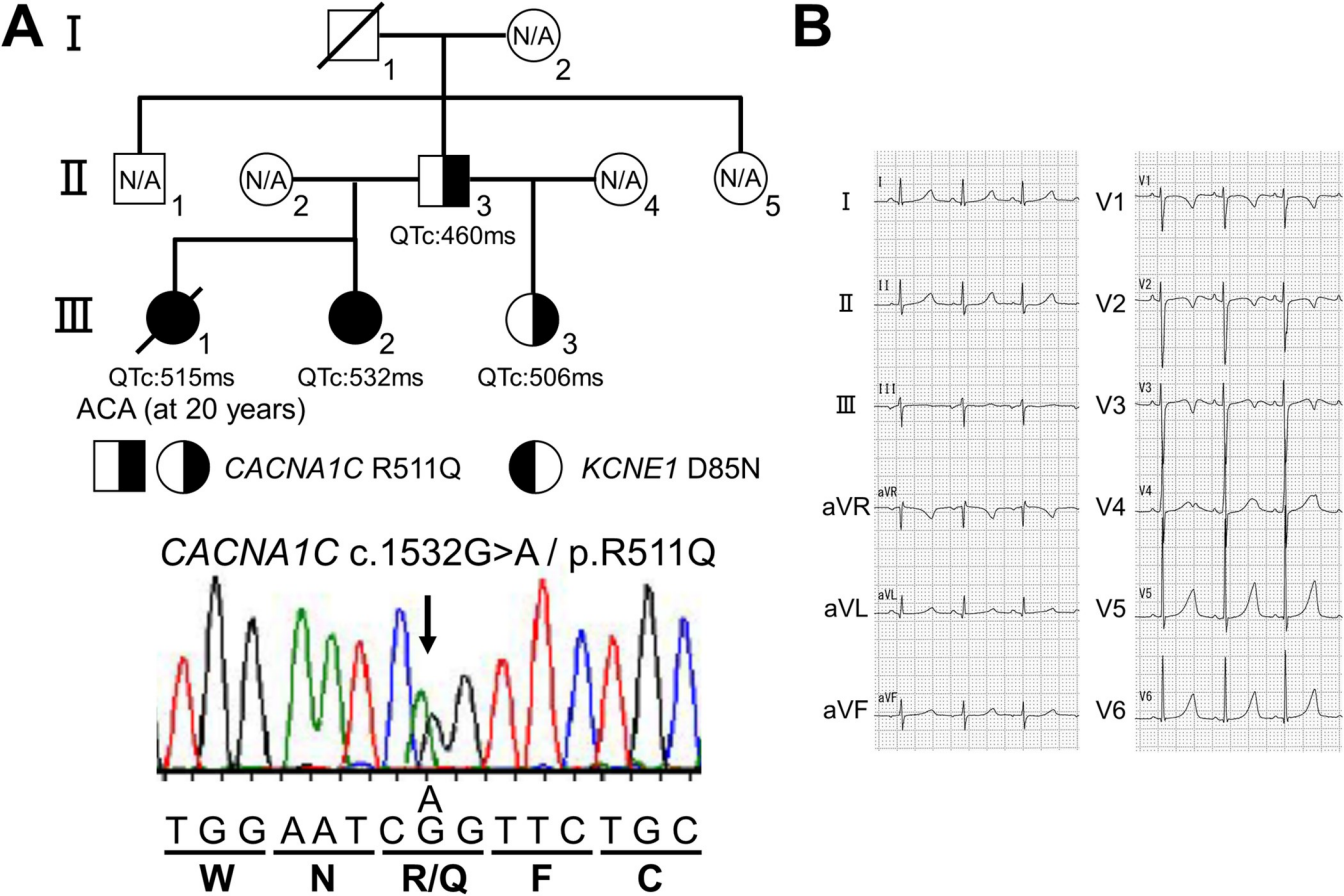
Identification of a *CACNA1C* R511Q mutation in a family with non-syndromic long QT syndrome. A: Pedigree of patients harboring the *CACNA1C* R511Q mutation (upper panel). Half black filled symbols indicate heterozygous carrier of *CACNA1C* R511Q alone, and full black filled symbols indicate heterozygous carrier of both *CACNA1C* R511Q and *KCNE1* D85N. ACA: aborted cardiac arrest, N/A: not genetically assessed. The electropherogram of a part of *CACNA1C* exon 12 of a patient (Ⅲ-3) is shown (lower panel). B: 12-lead ECG of the patient Ⅲ-3 in (A), recorded at 7 years old.

This study was approved by Gunma University Ethical Review Board for Medical Research Involving Human Subjects (approval number: 2017–15). The subjects of this study were 24 genotype-negative nsLQTS probands remaining after screening major LQTS-related genes (*KCNQ1*, *KCNH2*, *SCN5A*, *KCNE1* and *KCNE2*) using Sanger sequencing and three family members (Ⅱ-3, Ⅲ-2 and Ⅲ-3) of one proband (Ⅲ-1) (Fig 1A). Written informed consent for the genetic and functional analyses was obtained from the subjects. Clinical features of Ⅲ-1, Ⅲ-2 and Ⅱ-3 and genetic information of major LQTS-related genes (*KCNQ1*, *KCNH2*, *SCN5A*, *KCNE1* and *KCNE2*), obtained by Sanger sequencing, of Ⅲ-1 and Ⅲ-2 have already been reported [24].

A target panel sequencing of 72 genes, including LQTS-related genes, was employed in the subjects, as previously described [21]. Briefly, genomic DNA extracted from peripheral blood lymphocytes were subjected to custom panel sequencing using HaloPlex HS Custom (Agilent Technologies, Santa Clara, CA, USA) or xGen Predesigned Gene Capture Pools (Integrated DNA Technologies, Inc., Coralville, IA, USA) according to the manufacturer’s protocol. The resulting library was subjected to the paired-end sequencing of 150 bp reads on MiSeq (Illumina, San Diego, CA, USA). A bioinformatics analysis for HaloPlex HS Custom data was performed as previously described [21]. Reads obtained from xGen Predesigned Gene Capture Pools were subsequently aligned against hg19 using BWA and variant calling was performed using GATK in the BWA Enrichment v2.1.2 application on the BaseSpace Sequence Hub (Illumina) with default settings. The mean region coverage depth of 27 samples was 243 (150–426), and the percentage with target coverage of at least 20X was 98.8 (97.8–100). Among them, the mean region coverage depth was 327.9 (a *CACNA1C* R858H carrier), 196.4 (II-3), 204.8 (III-1), 290.1 (III-2) and 274 (III-3) (Fig 1A), and the percentage with target coverage of at least 20X was ≧99.9 in these cases. The identified variants, c.2573G>A/p.R858H in exon 19 and c.1532G>A/p.R511Q in exon 12 of *CACNA1C* (NM_000719.7), were validated by Sanger sequencing.

### Mutagenesis and heterologous expression

The human wild-type (WT) *CACNA1C* cDNA tagged by (EYFP)N_α1C,77_ in pcDNA vector and cDNAs of *CACNB2b* and *CACNA2D1*, both cloned in pcDNA3.1 vector, were kindly provided by Dr. Charles Antzelevitch (Masonic Medical Research Laboratory). Site-directed mutagenesis (*CACNA1C* R511Q) was performed using the QuikChangeⅡ Site-Directed Mutagenesis Kit (Agilent Technologies, Santa Clara, CA, USA) according to the manufacturer’s instructions. WT *CACNA1C* cDNA (2 μg) or R511Q *CACNA1C* cDNA (2 μg) in combination with *CACNB2b* cDNA (0.5 μg) and *CACNA2D1* cDNA (0.5 μg) was transiently transfected into tsA201 cells using Lipofectamine 2000 (Invitrogen, Carlsbad, CA, USA), and maintained in DMEM medium supplemented with 10% fetal bovine serum and 1% penicillin-streptomycin in a 5% CO_2_ incubator at 37°C for 36–48 hours before current recordings. Cells exhibiting green fluorescence were chosen for the current recordings.

### Electrophysiology

Membrane calcium or barium currents (I_Ca_ or I_Ba_) were recorded using whole-cell patch-clamp techniques at room temperature (23–25°C). Since electrophysiological studies of most gain-of-function *CACNA1C* mutations in the DⅠ-Ⅱ linker have been conducted under the condition of high extracellular calcium concentration, we also used a bath solution with a high calcium concentration. The bath solution contained (in mmol/L) 130 N-methyl-d-glucamine, 5 KCl, 15 CaCl_2_ (for I_Ca_) or BaCl_2_ (for I_Ba_), 1 MgCl_2_ and 10 HEPES (pH 7.35 with HCl), and the pipette solution contained (in mmol/L) 120 CsCl, 2 MgCl_2_, 2 MgATP, 5 CaCl_2_, 10 EGTA and 10 HEPES (pH 7.25 with CsOH). The electrode resistance ranged from 1.5 to 2.0 MΩ. Data acquisition was carried out using an Axopatch 200B amplifier and pCLAMP10.3 software (Molecular Devices, Sunnyvale, CA, USA). Currents were acquired at 20–50 kHz, and low pass-filtered at 5 kHz using an analog-to-digital interface (Digidata 1440A acquisition system, Molecular Devices). Current densities at each test potential were obtained by dividing the calcium currents by cell capacitance. The steady-state activation and steady-state inactivation curves were fitted with Boltzmann functions of the following forms: y = 1-1/{1+exp[(V_m_-V_1/2_)/*K*]} or y = 1/{1+exp[(V_m_-V_1/2_)/*K*]}, respectively, where y is the relative current, V_m_ is the membrane potential, V_1/2_ is the voltage at which half of the channels are available to open, and *K* is the slope factor. The time course of inactivation was fitted with a single or double exponential function of the following form: *I*(t)/*I*_max_ = A_0_+A_1_[1-exp(-t/τ)] or *I*(t)/*I*_max_ = A_0_+A_f_[1-exp(-t/τf)]+A_s_[1-exp(-t/τs)], where A and τ refer to the amplitudes and time constants, respectively, and f and s refer to the fast and slow components, respectively. *I* refers to the current, and t refers to the time. The time course of recovery from inactivation was fitted with a single exponential function of the following form: *I*(t)/*I*_max_ = A_0_+A_1_exp(-t/τ), as described previously [25, 26]. To avoid potential endogenous current contamination, recordings from the cells exhibiting peak inward current amplitudes of <0.3 nA were excluded from the analyses of the inactivation rate, steady-state inactivation and time courses of inactivation and recovery from inactivation.

### Statistical analysis

All data are expressed as mean ± standard error, and statistical comparisons were tested using the unpaired Student’s *t*-test with p<0.05 considered to be statistically significant. In some figures, the standard error bars are smaller than the data symbols.

## Results

### Identification of two *CACNA1C* mutations

We performed target panel sequencing in 24 genotype-negative nsLQTS probands after Sanger sequencing. As a result, we identified two *CACNA1C* mutations, an R858H mutation, which has already been reported, and a novel R511Q mutation, but did not detect any other pathogenic variants in LQTS-related genes [19]. Since the *CACNA1C* R858H mutation has already been functionally characterized by Fukuyama et al., we report the clinical characteristics of carriers of the novel *CACNA1C* R511Q mutation and its biophysical defects [19].

### Case presentations harboring the *CACNA1C* R511Q mutation

The index patient (Ⅲ-3) (Fig 1A), a 17-year-old female at the time of the genetic test, had been asymptomatic with QT prolongation that had first been identified at 7 years of age. She was a younger paternal half-sister of the deceased case (Ⅲ-1) (Fig 1A) we had previously reported [24]. Her ECG showed sinus rhythm with QT prolongation (HR: 98 bpm, QTc: 506 ms) (Fig 1B). An echocardiogram revealed no structural heart disease. Neither she nor any other family members had any cardiac or extra-cardiac abnormalities, except for QT prolongation. We first examined whether or not the index patient (Ⅲ-3) (Fig 1A) carried the *KCNE1* D85N variant, as with the proband (Ⅲ-1) (Fig 1A), using Sanger sequencing. However, we did not detect it, suggesting that other genetic factor(s) might be associated with this nsLQTS family.

In addition to the proband (Ⅲ-1) (Fig 1A), we therefore conducted target panel sequencing in the proband’s two sisters (Ⅲ-2 and Ⅲ-3) and father (Ⅱ-3) (Fig 1A) whose QT interval was slightly prolonged (HR: 60 bpm, QTc: 460 ms). As a result, we identified the *CACNA1C* R511Q variant in all four patients, and the finding was validated by Sanger sequencing (Fig 1A). The variant was present in neither gnomAD (https://gnomad.broadinstitute.org/) nor 8.3KJPN (https://jmorp.megabank.tohoku.ac.jp/202109/variants), and PolyPhen-2 (http://genetics.bwh.harvard.edu/pph2/) and SIFT (https://sift.bii.a-star.edu.sg/) indicated that the variant was probably damaging and deleterious, respectively (Table 1).

**Table 1:** Data from prediction software, database, ClinVar and our target panel sequencing of *CACNA1C* R511Q and *KCNE1* D85N variants.

	Prediction software		SNP database		ClinVar			Target panel sequencing Depth: Variant frequency			
AAChange.refGene	SIFT _pred	Polyphen2 _HDIV _pred	gnomAD _exome _EAS	8.3KJPN	Variation ID	CLNDN	CLNSIG	II-3	III-1	III-2	III-3
*CACNA1C*: NM_000719: exon12: c.G1532A: p.R511Q	Del	D	-	-	190641	Long_QT_syndrome| not_provided	Uncertain_ significance	223: 0.486	247: 0.498	342: 0.456	275: 0.433
*KCNE1*: NM_000219: exon4: c.G253A: p.D85N	Del	PD	0.0055	0.0113	13479	Cardiomyopathy| Long_QT_syndrome| Jervell_and_Lange-Nielsen_syndrome| Romano-Ward_syndrome| Long_QT_syndrome_5| Long_QT_syndrome_5,_ acquired,_susceptibility_to| Long_QT_syndrome_2/5| not_specified| Cardiovascular_phenotype| not_provided	Conflicting interpretations of pathogenicity, other, risk factor Benign(6);Likely benign(4);Uncertain significance(3)	-	121: 0.554	110: 0.482	-

D: damaging, Del: deleterious, PD: probably damaging.

ClinVar (https://www.ncbi.nlm.nih.gov/clinvar/variation/190641/) indicated that the variant was uncertain significance (Table 1). On the other hand, the *KCNE1* D85N variant was found in Ⅲ-1 and Ⅲ-2, as reported previously, but not in Ⅲ-3 and Ⅱ-3 (Table 1) (Fig 1A), suggesting that the *CACNA1C* R511Q variant might play a more important role in the generation of phenotypes than the *KCNE1* D85N variant [24].

### Biophysical defects of the *CACNA1C* R511Q mutation

**Fig 2:**
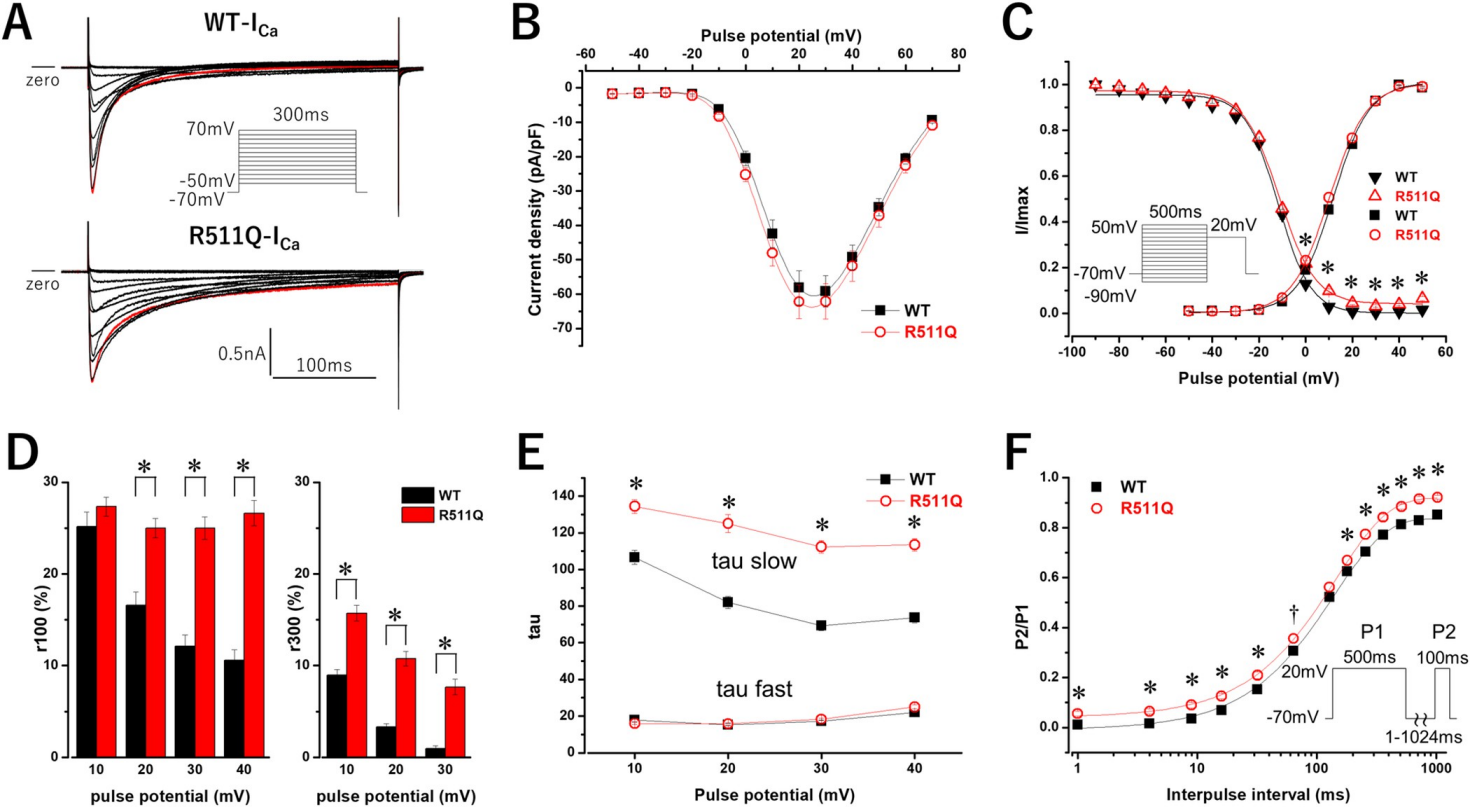
Expressed calcium currents (I_Ca_) of *CACNA1C* wild-type (WT-I_Ca_) and R511Q (R511Q-I_Ca_). A: Representative current tracings of WT-I_Ca_ (upper panel) and R511Q-I_Ca_ (lower panel) obtained by the pulse protocol shown in the inset A. Red lines indicate the currents when the depolarizing potential was +20 mV. B: The current-voltage relationship of WT-I_Ca_ (filled squares, n = 20) and R511Q-I_Ca_ (open red circle n = 18). Peak currents obtained by the pulse protocol were normalized to cell capacitances. C: The voltage dependence of activation of WT-I_Ca_ (filled squares, n = 20) and R511Q-I_Ca_ (open red circles, n = 18), and the voltage dependence of steady-state inactivation, obtained by the pulse protocol shown in the inset, of WT-I_Ca_ (filled reverse triangles, n = 12) and R511Q-I_Ca_ (open red triangles, n = 14). Plots were fitted with a Boltzmann function. Fitted data are shown in Table 2. *P<0.01 vs. WT-I_Ca_. D: The normalized amplitudes of persistent currents, assessed at 100-ms (r_100_) (left) and 300-ms (r_300_) (right) of various depolarizing potentials, of WT-I_Ca_ (n = 20) and R511Q-I_Ca_ (n = 18). Note that r_300_s at +40 mV were deleted because some r_300_s of WT-I_Ca_ at +40 mV were not precisely evaluated due to endogenous currents and/or leak currents. E: Fast and slow time constants (tau) of the voltage dependence of inactivation rate of WT-I_Ca_ (filled squares, n = 13) and R511Q-I_Ca_ (open red circles, n = 14). Inactivating currents obtained by the pulse protocol shown in the inset A were fitted by a double exponential function. *P<0.01 vs. WT-I_Ca_. F: The time course of recovery from inactivation of WT-I_Ca_ (filled squares, n = 7) and R511Q-I_Ca_ (open red circles, n = 9). Plots were fitted with a single exponential function. ^†^P<0.05 vs. WT-I_Ca_, *P<0.01 vs. WT-I_Ca_.

To examine the pathogenicity of the *CACNA1C* R511Q variant, we conducted a functional analysis by measuring whole-cell membrane currents using Ca^2+^ as a charge carrier (I_Ca_). As shown in Fig 2A, WT CACNA1C exhibited I_Ca_ (WT-I_Ca_), and R511Q CACNA1C exhibited I_Ca_ (R511Q-I_Ca_) resembling WT-I_Ca_. The peak current density, measured at +20 mV from a holding potential of -70 mV, and steady-state activation of R511Q-I_Ca_ were comparable to those of WT-I_Ca_ (Table 2) (Fig 2B and 2C).

**Table 2:** Parameters of steady-state activation and steady-state inactivation for WT-I_Ca_ and R511Q-I_Ca_.

		Steady-state activation		Steady-state inactivation	
	Current density (pA/pF) at +20mV	V_1/2_ (mV)	*K* (mV)	V_1/2_ (mV)	*K* (mV)
WT	58±5.0 (n = 20)	12.0±0.7	7.6±0.1	-11.7±0.6 (n = 12)	7.0±0.2
R511Q	62±4.9 (n = 18)	10.4±0.7	8.0±0.2	-11.1±0.4 (n = 14)	7.9±0.3*

V_1/2_: voltage at which half of the channels are available to open, *K*: slope factor

*P<0.01 vs. WT.

However, residual currents at 100-ms (r_100_) of depolarization potentials (+20 mV, +30 mV and +40 mV) in R511Q-I_Ca_ were significantly larger than those in WT-I_Ca_ (Table 3) (Fig 2D). Notably, although r_100_s of WT-I_Ca_ decreased with higher depolarization potentials, those of R511Q-I_Ca_ were maintained. On the other hand, residual persistent currents at 300-ms (r_300_) of depolarizing potentials (+10 mV, +20 mV and +30 mV) in R511Q-I_Ca_ were significantly larger than those in WT-I_Ca_ (Table 3) (Fig 2D). Although r_300_s of WT-I_Ca_ markedly decreased with higher depolarization potentials, those of R511Q-I_Ca_ showed a mild decrease. Thus, the difference in residual currents between WT-I_Ca_ and R511Q-I_Ca_ became larger with higher depolarization potentials.

**Table 3:** Parameters of persistent currents and inactivation for WT-I_Ca_ and R511Q-I_Ca_.

pulse potential (mV)		persistent currents		inactivation			
		r_100_ (%)	r_300_ (%)	A fast	tau fast (ms)	A slow	tau slow (ms)
+10	WT	25.2±1.6	9.0±0.6	0.49±0.01	17.9±0.9	0.51±0.01	105.4±3.8
	R511Q	27.4±1.0	15.7±0.9*	0.55±0.02*	15.9±0.5	0.45±0.02*	134.4±3.8*
+20	WT	16.6±1.5	3.3±0.3	0.52±0.01	15.3±0.6	0.48±0.01	81.3±3.3
	R511Q	25.0±1.1*	10.8±0.8*	0.49±0.02	15.8±0.6	0.51±0.02	125.1±5.0*
+30	WT	12.1±1.3	0.9±0.3	0.55±0.02	17.2±0.8	0.45±0.02	67.9±2.6
	R511Q	25.0±1.3*	7.7±0.8*	0.43±0.01*	18.3±0.7	0.57±0.01*	112.3±3.4*
+40	WT	10.6±1.1	-	0.59±0.02	22.0±0.9	0.41±0.02	72.3±2.9
	R511Q	26.6±1.4*	-	0.39±0.02*	25.0±1.2	0.61±0.02*	113.4±3.4*

r_100_ and r_300_ indicate residual persistent currents (%) at 100-ms and 300-ms of depolarizing potential, respectively. Note that r_300_s at +40 mV were deleted because some r_300_s of WT-I_Ca_ at +40 mV were not precisely evaluated due to endogenous currents and/or leak currents. A fast and A slow indicate the amplitudes of fast and slow components of inactivation, respectively.

*P<0.01 vs. WT at each test potential.

I_Ca_ displays two forms of inactivation: voltage-dependent inactivation (VDI) and calcium-dependent inactivation (CDI) [3, 27, 28]. CDI and VDI are thought to correspond to the fast and slow component of inactivation, respectively. Inactivating currents of WT-I_Ca_ and R511Q-I_Ca_ during 300-ms depolarizing potentials from +10 mV to +40 mV could be fitted by a double exponential function. The time constants of fast components of R511Q-I_Ca_ were comparable to those of WT-I_Ca_ at each test potential, although the amplitudes of fast components of R511Q-I_Ca_ became smaller than those of WT-I_Ca_ as the depolarizing potentials became higher (Table 3) (Fig 2E). In contrast, the time constants of slow components of R511Q-I_Ca_ were significantly larger than those of WT-I_Ca_ at each test potential, and the amplitudes of slow component of R511Q-I_Ca_ became larger than those of WT-I_Ca_ as the depolarizing potentials became higher (Table 3) (Fig 2E). These findings suggest that, in R511Q-I_Ca_, the CDI decreased and VDI increased with slower inactivation, especially at higher depolarization potentials.

The steady-state inactivation was assessed using a pulse protocol shown in the inset in Fig 2C. The voltage at which half of the channels are available to open (V_1/2_) of R511Q-I_Ca_ was comparable to that of WT-I_Ca_ (Table 2) (Fig 2C). However, the slope factor (*K*) of R511Q-I_Ca_ was significantly larger than that of WT-I_Ca_ (Table 2) (Fig 2C), and the I/I_max_ of R511Q-I_Ca_ was significantly larger than that of WT-I_Ca_ when prepulse potentials were between 0 mV and +50 mV (Fig 2C), possibly due to larger persistent currents during prepulse potentials in R511Q-I_Ca_, which resulted in increased window currents in R511Q-I_Ca_ in comparison to those in WT-I_Ca_ (Fig 2C).

The recovery from inactivation was assessed using a double pulse protocol shown in the inset in Fig 2F, and plots were fitted by a single exponential function (Fig 2F). Although the P2/P1 of R511Q-I_Ca_ was significantly larger than that of WT-I_Ca_ from the beginning of an interpulse duration of several ms, possibly due to larger persistent currents in R511Q-I_Ca_ during P1 pulses, the time constant of R511Q-I_Ca_ was comparable to that of WT-I_Ca_ (WT-tau: 135±3.7 ms, n = 7, R511Q-tau: 145±7.6 ms, n = 9, P = NS). This indicated that the time course of recovery from inactivation of R511Q-I_Ca_ was not markedly different from that of WT-I_Ca_.

**Fig 3:**
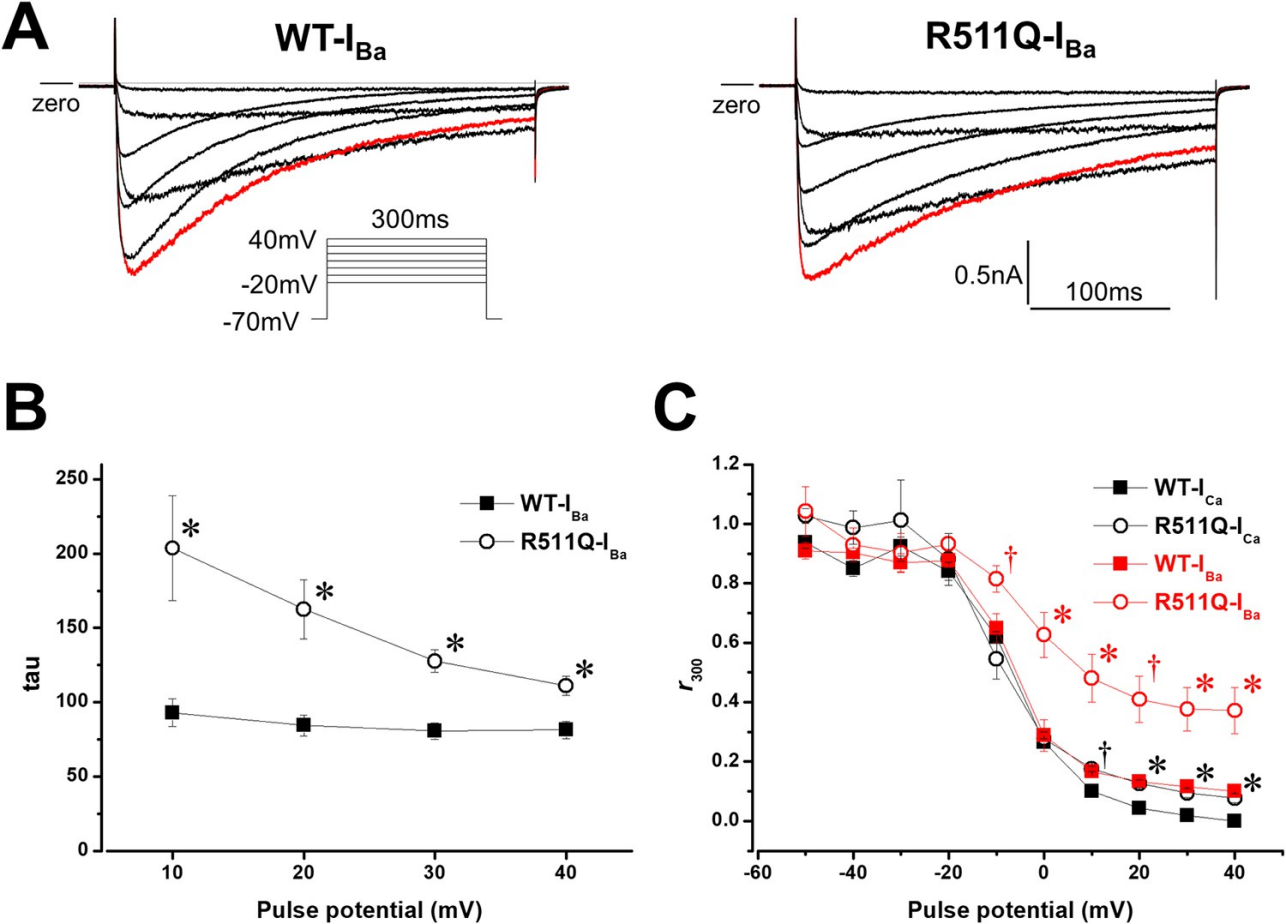
Barium currents (I_Ba_) of *CACNA1C* WT (WT-I_Ba_) and R511Q (R511Q-I_Ba_). A: Representative current tracings of WT-I_Ba_ (left panel) and R511Q-I_Ba_ (right panel), obtained by the pulse protocol shown in the inset. Red lines indicate the currents when the depolarizing potential was +10 mV. B: Time constants (tau) of the voltage dependence of inactivation rate of WT-I_Ba_ (filled squares, n = 5) and R511Q-I_Ba_ (open circles, n = 4). Inactivating currents obtained by the pulse protocol shown in the inset A were fitted by a single exponential function. *P<0.01 vs. WT-I_Ba_. C: Remaining currents after 300-ms depolarization normalized to peak currents (*r*_300_) of WT-I_Ca_ (filled squares, n = 5), R511Q-I_Ca_ (open circle, n = 4), WT-I_Ba_ (filled red squares, n = 5) and R511Q-I_Ba_ (open red circles, n = 4) at various potentials are shown. ^†^P<0.05 vs. WT-I_Ca_, *P<0.01 vs. WT-I_Ca_, ^†^(red)P<0.05 vs. WT-I_Ba_, *(red)P<0.01 vs. WT-I_Ba_.

The VDI can be studied with Ba^2+^ as the charge carrier, which excludes the CDI process [28]. Therefore, the Ca^2+^ in the bath solution was replaced with Ba^2+^, and then Ba^2+^ currents through calcium channels (I_Ba_) were recorded. As shown in Fig 3A, WT-I_Ba_ and R511Q-I_Ba_ exhibited delayed inactivation compared to WT-I_Ca_ and R511Q-I_Ca_, respectively. Inactivating currents of WT-I_Ba_ and R511Q-I_Ba_ during depolarizing potentials from +10 mV to +40 mV could be fitted by a single exponential function. The time constants of R511Q-I_Ba_ were still larger than those of WT-I_Ba_ at each test potential (Fig 3B), suggesting that the VDI rather than the CDI of R511Q might be impaired. The VDI was presented as the fraction of current remaining after a 300-ms depolarization normalized to peak (*r*_300_) across various voltages, and the extent of CDI was calculated as *f*_300_ = (*r*_300Ba_-*r*_300Ca_)/*r*_300Ba_ [18]. The VDI of R511Q-I_Ba_ and R511Q-I_Ca_ was significantly delayed compared to that of WT-I_Ba_ and WT-I_Ca_, respectively (Fig 3C). However, the *f*_300_ for WT at +20 mV (WT-*f*_300_) and *f*_300_ for R511Q at +20 mV (R511Q-*f*_300_) were 0.67±0.05 (n = 5) and 0.67±0.05 (n = 4), respectively (p = NS). Taken together, these results indicated that the VDI of R511Q was predominantly impaired compared to the CDI.

## Discussion

### Identification of two *CACNA1C* mutations associated with nsLQT8

We identified two *CACNA1C* mutations, using target panel sequencing, in 24 genotype-negative nsLQTS patients after screening of major LQTS-related genes using Sanger sequencing. This appears to be consistent with the notion that nsLQT8 is more prevalent than previously expected [19, 20, 29, 30]. Therefore, the *CACNA1C* should be involved in genes for screening LQTS patients.

### *CACNA1C* mutations that have been associated with QT prolongation

Cav1.2 is encoded by *CACNA1C* and composed of four homologous but non-identical domains (DⅠ-DⅣ) and intracellular N- and C-termini. Each domain contains six transmembrane spanning segments (S1-S6) consisting of a voltage-sensing domain (VSD) and pore-forming domain, intracellular and extracellular loops [28]. A gain-of-function of I_Ca_ by *CACNA1C* mutations throughout the Cav1.2 structure has been associated with sLQTS (TS, aTS and COTS) or nsLQT8.

A *CACNA1C* G406R mutation in exon 8A (G406R-8A), located in the DⅠ-Ⅱ linker, was firstly identified in type-1 TS (TS1) [7]. Next, *CACNA1C* G402S and G406R mutations in exon 8 (G402S-8 and G406R-8, respectively), located in the DⅠS6 or DⅠ-Ⅱ linker, were also identified in type-2 TS (TS2) [8]. Later, other *CACNA1C* mutations associated with aTS, COTS and nsLQT8 were identified [9–20, 29].

Gain-of-function of *CACNA1C* mutations associated with QT prolongation can be caused by multiple mechanisms, including delayed inactivation, increased persistent currents, increased window currents, increased current density, a combination thereof and increased permeability of non-selective monovalent cations [14]. However, the mechanisms that cause other cardiac and extra-cardiac phenotypes remain unknown.

### Novel *CACNA1C* R511Q mutation and its biophysical defects

We identified the *CACNA1C* R511Q mutation, located in the DⅠ-Ⅱ linker, in four patients in one LQTS family. All patients harboring the mutation exhibited QT prolongation but no other cardiac or extra-cardiac phenotypes.

A functional study using a heterologous expression system revealed that the *CACNA1C* R511Q displayed a delay of the slow component of I_Ca_ inactivation, increased persistent currents, and increased window currents, without a change of the current density.

The inactivation of I_Ca_ occurs via two mechanisms: the VDI, which is linked to the change of transmembrane potential, and the CDI, which is mediated by calcium ions that carry the current [3, 27, 28]. The molecular determinants of the VDI include the cytoplasmic ends of the S6 segments, DⅠ-Ⅱ linker, and C-terminus of Cav1.2 [22]. In particular, the DⅠ-Ⅱ linker has been suggested to be a particle that occludes the channel pore during inactivation [22, 23]. In contrast, the molecular determinants of the CDI include C-terminus [22, 28, 31, 32].

When Ba^2+^ is used as a charge carrier (I_Ba_), the fast component of inactivation is lost and the slow component of inactivation is markedly decelerated [28]. In our experiments, inactivating Ba^2+^ currents in both WT-I_Ba_ and R511Q-I_Ba_ could be fitted by a single exponential function rather than a double exponential function, possibly due to a loss of the fast component of I_Ca_ inactivation. Time constants of inactivating currents in R511Q-I_Ba_ were still larger than those in WT-I_Ba_, indicating that the VDI of R511Q-I_Ca_ was predominantly decelerated compared to that of WT-I_Ca_. Furthermore, the extent of calculated CDI was not different between WT and R511Q, which also indicated that the VDI of R511Q-I_Ca_ was predominantly decelerated.

### *CACNA1C* mutations in the DⅠS6 or DⅠ-Ⅱ linker

Focusing on mutations in the DⅠS6 and DⅠ-Ⅱ linker, those for TS1 and TS2 (G406R-8A, G402S-8 and G406R-8) displayed a marked delay of I_Ca_ inactivation, marked increases of persistent currents and window currents (Table 4) [7, 8]. A G419R mutation for aTS displayed an increased current density with accelerated inactivation (Table 4) [17]. Mutations, R518H and R518C for COTS, displayed a delay of I_Ca_ inactivation, increased persistent currents and increased window currents (but those were weaker than TS mutations: G406R-8A, G402S-8 and G406R-8) with a reduced current density (Table 4) [18]. In our study, the R511Q mutation displayed a delay of I_Ca_ inactivation, increased persistent currents, which is very likely the main actor of QT prolongation, and increased window currents (but those were weaker than TS mutations: G406R-8A, G402S-8 and G406R-8) without a change of current density. These biophysical defects of the R511Q mutation were more modest than seen in other mutations for TS, aTS or COTS (Table 4), which may be the reason why the R511Q mutation is associated with nsLQT8 but not with other cardiac and extra-cardiac phenotypes. Otherwise, TS mutations have been reported to impair not only the VDI but also the CDI, suggesting that an impairment of the CDI may be associated with the manifestation of other cardiac or extra-cardiac phenotypes [7, 33–35]. These findings provide a novel insight into the pathophysiological roles of the DⅠ-Ⅱ linker in phenotypic manifestations.

**Table 4:** Comparison of electrophysiological parameters of gain-of-function *CACNA1C* mutations in DⅠ-Ⅱ linker that have been reported.

Phenotype	TS1	TS2		aTS	COTS		nsLQTS
Mutation	G406R-8A	G402S-8	G406R-8	G419R	R518C	R518H	R511Q
Expressed cells	CHO cells	Xenopus oocytes	Xenopus oocytes	HEK293T cells	HEK293 cells	HEK293 cells	tsA201 cells
Current density	no change	no change	no change	increased (4-fold)	decreased (55.6%)	decreased (63.2%)	no change
Inactivation rate	delayed (marked)	delayed (marked)	delayed (marked)	accelerated (slight)	delayed	delayed	delayed
Persistent currents	increased (marked)	increased (marked)	increased (marked)	no change	increased (~7.0-fold)	increased (~6.6-fold)	increased
SSA	no change	no change	negatively shifted	negatively shifted (~10 mV)	no change	positively shifted (~4.5 mV)	no change
SSI	positively shifted (marked)	positively shifted (marked)	positively shifted (marked)	no change	positively shifted (~6.8 mV)	positively shifted (~7.0 mV)	not shifted
Window currents	increased (marked)	increased (marked)	increased (marked)	increased	increased	increased	increased (slight)
References	[7]	[8]	[8]	[17]	[18]	[18]	this study

TS1, type-1 Timothy syndrome; TS2, type-2 Timothy syndrome; aTS, atypical Timothy syndrome; COTS, cardiac only Timothy syndrome; nsLQT8, non-syndromic long QT syndrome, SSA, steady-state activation: SSI, steady-state inactivation.

Korkosh et al. recently built structural models of Cav1.2 and proposed mechanisms underlying the VDI: The cytoplasmic N-terminal part of VSDII (DIIS0) in the DⅠ-Ⅱ linker is bound with the α1-interaction domain (AID). Following voltage-dependent channel activation, the cytoplasmic face of DIIS0 would perturb and shift DIIS0-bound AID toward the pore axis. The AID-linked DIS6 would bend at the flexible G402 and G406, facilitating the activation-gate closure and thus the VDI [36]. The R511 position in DIIS0 may form a salt bridge with acidic residues in positions immediately C-terminal to the AID. Therefore, the R511Q mutation would destroy the salt bridges and weaken the DIIS0-AID contact, retarding the AID displacement thus delaying the VDI. Regarding the interaction of AID and β-subunit, four basic residues (R514, R515, R518 and K522) in DIIS0 are thought to provide large contributions to the interaction energy between AID and β-subunit, while R511 is not [36]. Further studies are required to reveal whether or not R511Q affects the DIIS0-AID-β-subunit interaction.

TS patient- and COTS patient-derived human induced pluripotent stem cell-derived cardiomyocytes (hiPSC-CMs) have been established [37, 38]. An analysis of patient (with the *CACNA1C* R511Q mutation)-derived hiPSC-CMs and mutant allele-specific knockout using a clustered regularly interspaced short palindromic repeats (CRISPR)-CRISPR associated protein 9 (CRISPR-Cas9) system may reveal the pathophysiological roles of the *CACNA1C* R511Q in QT prolongation and arrhythmogenesis.

### Other genetic variants in this nsLQTS family

All four patients presenting with QT prolongation in the family carried the *CACNA1C* R511Q mutation, but not all carried the *KCNE1* D85N variant which was reported to be a disease-causing or a drug-induced LQTS variant [24, 39, 40]. This suggested that the *CACNA1C* R511Q mutation might be a main contributor to QT prolongation in this family, and the *KCNE1* D85N variant may have contributed less to QT prolongation in comparison to the *CACNA1C* R511Q mutation. Although the father (Ⅱ-3) (Fig 1A) harbors the *CACNA1C* R511Q mutation, his QTc interval was only slightly prolonged. This may be due to the fact that the QTc interval in males is shorter than that in females. The contribution of these variants to QT prolongation and cardiac events in this family needs to be further investigated.

## Conclusions

Target panel sequencing in 24 genotype-negative nsLQTS patients after Sanger screening identified two *CACNA1C* mutations: An R858H mutation in one proband and a novel R511Q mutation in one family. This supported the notion that nsLQT8 is more prevalent than previously expected. Despite the fact that *CACNA1C* mutations in the DⅠ-Ⅱ linker have been associated with sLQTS (TS, aTS and COTS), the R511Q mutation in this site is associated with nsLQT8. The biophysical defects of the R511Q mutation were a delay of slow component of I_Ca_ inactivation through predominant impairment of the VDI, increased persistent currents, and increased window currents, without a change of the current density. The degree of functional impairment associated with the R511Q was more modest in comparison to that seen with TS, aTS and COTS mutations. These findings provide novel insights into the structure-function relationships of Cav1.2 and the pathophysiological roles of the DⅠ-Ⅱ linker in phenotypic manifestations.

## Abbreviations

AID: α1-interaction domain
aTS: atypical Timothy syndrome
CDI: calcium-dependent inactivation
COTS: cardiac only Timothy syndrome
DⅠ-Ⅱ: domain Ⅰ-Ⅱ
I_Ba_: barium currents
I_Ca_: calcium currents
LQTS: long QT syndrome
nsLQT8: non-syndromic type-8 long QT syndrome
sLQT8: syndromic type-8 long QT syndrome
TS: Timothy syndrome
VDI: voltage-dependent inactivation
WT: wild-type
